# Reduced abundance of *Faecalibacterium prausnitzii* in the gut microbiota of children diagnosed with cancer, a pilot study

**DOI:** 10.3389/frmbi.2023.1151889

**Published:** 2023-11-07

**Authors:** Eric J. de Muinck, Pål Trosvik, Nga Nguyen, Peter J. Fashing, Vetle M. Stigum, Nina Robinson, Johanne U. Hermansen, Monica C. Munthe-Kaas, Lars O. Baumbusch

**Affiliations:** ^1^ Centre for Ecological and Evolutionary Synthesis (CEES), Department of Biosciences, University of Oslo, Oslo, Norway; ^2^ Department of Anthropology and Environmental Studies Program, California State University Fullerton, Fullerton, CA, United States; ^3^ Department of Oncology, Division of Pediatric and Adolescent Medicine, Oslo University Hospital Rikshospitalet, Oslo, Norway; ^4^ Department of Pediatric Research, Division of Paediatric and Adolescent Medicine, Oslo University Hospital Rikshospitalet, Oslo, Norway

**Keywords:** cancer, children, fecal, gastrointestinal, gut, microbiome, microbiota Norway

## Abstract

**Background:**

There is an increasing awareness of the importance of the gut microbiome in disease progression and the maintenance of human health. However, links between the microbiome and cancer onset remain relatively unexplored. This is especially the case for childhood cancers, which although rare, are the predominant cause of death among children in Western countries.

**Methods:**

Fecal samples were collected from patients, before the onset of treatment, by the Norwegian Childhood Cancer Biobank in Oslo and from children attending kindergartens in Oslo, Norway. Using 16S rRNA gene amplicon sequencing, we compared the gut microbiome compositions of the children diagnosed with cancer with children attending kindergarten.

**Results:**

We observed significant differences in the relative abundances of several taxa, including a striking depletion of *Faecalibacterium prausnitzii*, an important taxa linked to gut health maintenance.

**Conclusions:**

Our observations provide evidence that the gut microbiome may play an important role in physiological changes associated with the onset of childhood cancer. However, further studies should be designed in order to validate our findings. Furthermore, these results suggest that variations in the microbial community could potentially be used as an early indicator of childhood cancer.

## Introduction

The adult human gastrointestinal microbiome is a stable and complex community comprised of trillions of microorganisms (Cho and Blaser, 2012). Humans are born with an essentially sterile gastrointestinal tract, with microbial colonization starting during birth, and stabilizing into an adult-like composition around three years of age (Milani et al., 2017; Kennedy et al., 2023). The establishment of the microbial community in infants has become widely recognized as a fundamental developmental process (Bokulich et al., 2016; Tamburini et al., 2016; Chu et al., 2017; Milani et al., 2017).

The human gut microbiome plays several essential roles for health and physiology of the host, including influencing the development of the immune system, protection from pathogens, and the production of vitamins and short-chain fatty acids. It has been suggested by (Levy et al., 2017), that the gut microbiome may be influenced by host factors that could be used to indicate gut dysbiosis. In the case of Ulcerative colitis (UC) and Crohn’s disease (CD), shifts in the bacterial community with depletion of *Faecalibacterium prausnitzii* have been found (Zhang et al., 2016) and similar shifts have been observed in colon cancer (Lopez-Siles et al., 2016).

The link between cancer and the microbiome may be causal, as some microbes are considered oncogenic (Chang and Parsonnet, 2010). However, it is still unclear whether the general composition of the gut microbiota can influence the development of cancer (Zitvogel et al., 2017), e.g. if certain microbial community profiles increase individual susceptibility to the disease. It is also possible that the composition of the gut microbiota is altered by changes in the host physiology resulting from or by cancer, with important implications for host health and treatment prospects.

Childhood cancer is still the predominant cause of death among children over a year in Western countries despite significantly improved treatment and increased knowledge of the molecular mechanisms of various cancers (Siegel et al., 2021). Pediatric cancers are entirely different in comparison to adult cancers, in terms of frequencies, clinical behavior, and the potential factors involved in the onset of the disease. Current knowledge about the role of the gut microbiome in childhood cancer is scarce (Bai et al., 2018). It has, however, been shown that the gastrointestinal microbial community differs between pediatric versus adolescent Acute Lymphomatic Leukemia (ALL) patients in comparison to a healthy control group at the time of diagnosis (Rajagopala et al., 2016), indicating that the gastrointestinal microbiome may play a role in the establishment and progression of the disease. Further, it has been speculated that in pediatric acute leukemia (AL), the perturbation of the gut microbiome may extend the actual treatment with antibiotics, chemo- or immune therapeutics and thus, contribute to long term side-effects in AL survivors (Masetti et al., 2021).

To our knowledge, this pilot study is one of the first studies investigating the gut microbiota of children diagnosed with cancer (D’Amico et al., 2022; Masetti et al., 2022 Cancers). We collected fecal samples from patients diagnosed with cancer prior to the beginning of the treatment regime. In addition, as normal controls, we obtained fecal samples from children attending two different kindergartens in Oslo. We performed 16S rRNA gene amplicon sequencing of the DNA extracted from fecal samples in order to look for differences in the gut microbiota composition between children diagnosed with cancer and a group of healthy children.

## Methods

### Participants

Fecal samples from children diagnosed with cancer (N=48) were collected (prior to treatment) at the hospital and frozen directly upon sampling, without any kind of preservation medium (**Figure S1**). Samples were then withdrawn from the Norwegian Childhood Cancer Biobank (NCCB), where biological samples are stored according to standard protocols (Hermansen et al., 2021). Sample collection began in March 2017 and was ongoing until DNA extraction and sequencing in 2020. After removal of samples due to labeling issues and low read counts, 33 patients remained. Of these, 12 had been diagnosed with tumors of the central nervous system (CNS), 13 with solid tumors (ST), and eight with leukemia (**Figure S1**; **Table 1**).

**Table 1:** Overview of the clinical information of the patients.

ID	Age at diagnosis (years)	Diagnosis group	Gender	Attending daycare
1	15	Solid tumor	male	unknown
2	0	Solid tumor	male	no
8	16	Liquid malignancy	female	yes
16	3	CNS	male	yes
17	17	CNS	female	unknown
20	3	CNS	female	yes
21	2	CNS	male	yes
22	1	Liquid malignancy	female	no
42	1	Solid tumor	male	yes
53	16	Solid tumor	male	unknown
84	11	CNS	male	yes
87	17	CNS	male	yes
93	0	Solid tumor	female	no
111	4	Liquid malignancy	male	yes
137	6	CNS	male	unknown
162	11	Solid tumor	male	yes
181	0	Solid tumor	male	no
184	0	Benign solid tumor*	male	no
185	2	Liquid malignancy	male	yes
191	2	Solid tumor	male	no
194	0	Solid tumor	female	no
195	6	Solid tumor	male	unknown
196	8	Solid tumor	female	yes
302	11	Liquid malignancy	female	yes
306	5	Liquid malignancy	male	unknown
308	11	Solid tumor	female	yes
310	1	Solid tumor	male	yes
315	15	CNS	male	yes
322	15	Solid tumor	male	yes
324	5	Liquid malignancy	male	yes
326	4	Liquid malignancy	female	unknown
329	13	CNS	male	yes
330	8	CNS	male	yes

Fecal samples from healthy children were collected at home by the parents or at kindergarten by the teachers into tubes containing 95% ethanol as part of another study during the first half of 2020, in order to investigate the effects of environmental exposure on the microbiome in children. Sample storage for these samples was between seven and eight months before DNA extraction. One subpopulation included children (age 3-5 years) attending a traditional Norwegian kindergarten (n=10) and the other subpopulation was based on samples from children spending their time in a forest kindergarten (age 2-5 years). A major factor that differentiates a traditional kindergarten from a forest kindergarten is the amount of time spent outdoors in closer contact with nature and its natural variety of bacteria. Both populations of children (n=15) resided in Oslo and exhibited no obvious signs of physical ailments or cognitive deficits. In order to test for systematic bias due to sampling, three fecal samples from three individuals were divided into aliquots that were frozen with equivalent aliquots stored in ethanol (18 samples in total). For one individual, two of the frozen samples did not amplify. For that individual we only include one frozen and one ethanol stored sample in the analysis. (14 samples total, 7 frozen and 7 stored in ethanol).

### DNA extraction and sequencing

Fecal DNA was extracted using the MagAttract PowerSoil DNA KF kit (Qiagen) according to the manufacturer’s instructions. Library preparation for DNA sequencing was carried out as previously described (de Muinck et al., 2017), targeting the V4 region of the 16S rRNA gene with the 515f-806r primer pair. A 2x300 paired-end sequencing reaction was performed using the MiSeq platform at the Norwegian Sequencing Centre.

### Data analysis and statistics

Sequence read demultiplexing was carried out using a custom routine developed at NSC (https://github.com/nsc-norway/triple_index-demultiplexing). Further sequence data processing was performed using the Divisive Amplicon Denoising Algorithm as implemented in the dada2 v1.16 R-package (Callahan et al., 2016). All dada2 settings were default except for the filterAndTrim function where truncLen was set to 240 for read one and 160 for read two, and the max EE argument was set to two for read one and two for read two. Nine samples were excluded from further analysis in the childhood cancer cohort due to labeling issues, six were removed due to low read numbers, leaving 33 samples for analyses (**Figure S1**; **Table 1**). The total sum of sequences for these samples, after filtering, pair merging, and chimera removal, was 3,428,605 (mean 59,114 ± 29,457 s.d.). Taxonomic classification of amplicon sequence variants (ASVs) was done using the Ribosomal Database Project v16 training set (Cole et al., 2014). Singleton ASVs were removed from the data, as were ASVs that did not receive a reliable taxonomic assignment at the phylum level. This resulted in a total of 1224 ASVs (mean 124 ± 48 s.d.).

The following R packages were used: PERMANOVA was carried out using the adonis2 function in the Vegan v2.5.6, using Bray-Curtis distances. The random forest model was computed using random Forest v4.6.14 (Ho, 1995). Significance of the random forest model was calculated using the rfUtilities v2.1.5. Differential occurrence analysis of ASVs between the patient and control group based on the negative binomial distribution was carried out using DESeq2 v1.28.1 (Love et al., 2014), with reported p-values subjected to Benjamini-Hochberg correction for multiple hypothesis testing. For DESeq2 testing, we wanted to focus on prevalent ASVs that occurred in several individuals. Therefore, the count data was filtered to only include ASVs that occurred at a minimum of 100 sequence reads in a minimum of five individuals, leaving 133 ASVs.

## Results

Of the 33 patients included in the final analyses, 12 had been diagnosed with tumors of the central nervous system (CNS), 13 with solid tumors (ST), and eight with leukemia (**Table 1**). The cohort of patients included children within an age range from newborn to 17 years. In particular in the ST group was the median age two years (inter quartile range 0-11) relative to 4.5 (3.5-6.5) for leukemia and 9.5 (3-15) for CNS tumors. A PERMANOVA model did not find any significant effect of sex (p=0.95). However, non-metric multidimensional scaling (NMDS) analysis demonstrated the large effect age had on the microbiota in this cohort (**Figure S2**). Yatsunenko et al. (2012) and others (Stewart et al., 2018) have reported that the gut microbiota develops an adult composition by three years of age. Thus, we divided the patients into those less than three years old and those three years or older. A PERMANOVA model including both, diagnosis and age group, did not demonstrate any significant effects on diagnosis (p=0.27), while age group was highly significant (p<0.001).

Diversity was significantly reduced (**Figures S3A, B**) in the patients younger than three years, both relative to those three years and older and healthy controls three years and older (henceforth referred to as controls), in terms of species richness and Shannon entropy (p<0.002 for all comparisons, Wilcoxon rank sum test). This result was in line with the expectations (e.g. Yatsunenko et al., 2012). In order to carry out a more realistic comparison with our kindergarten controls, we excluded children younger than three years (11 in total) from our patient cohort in the following analyses. Within the patients of age three years and over, there was no significant effect of age on the microbiota (p=0.57, PERMANOVA). Furthermore, all patients will henceforth be treated as a single group in the comparisons with the control group.

**Figure 1:**
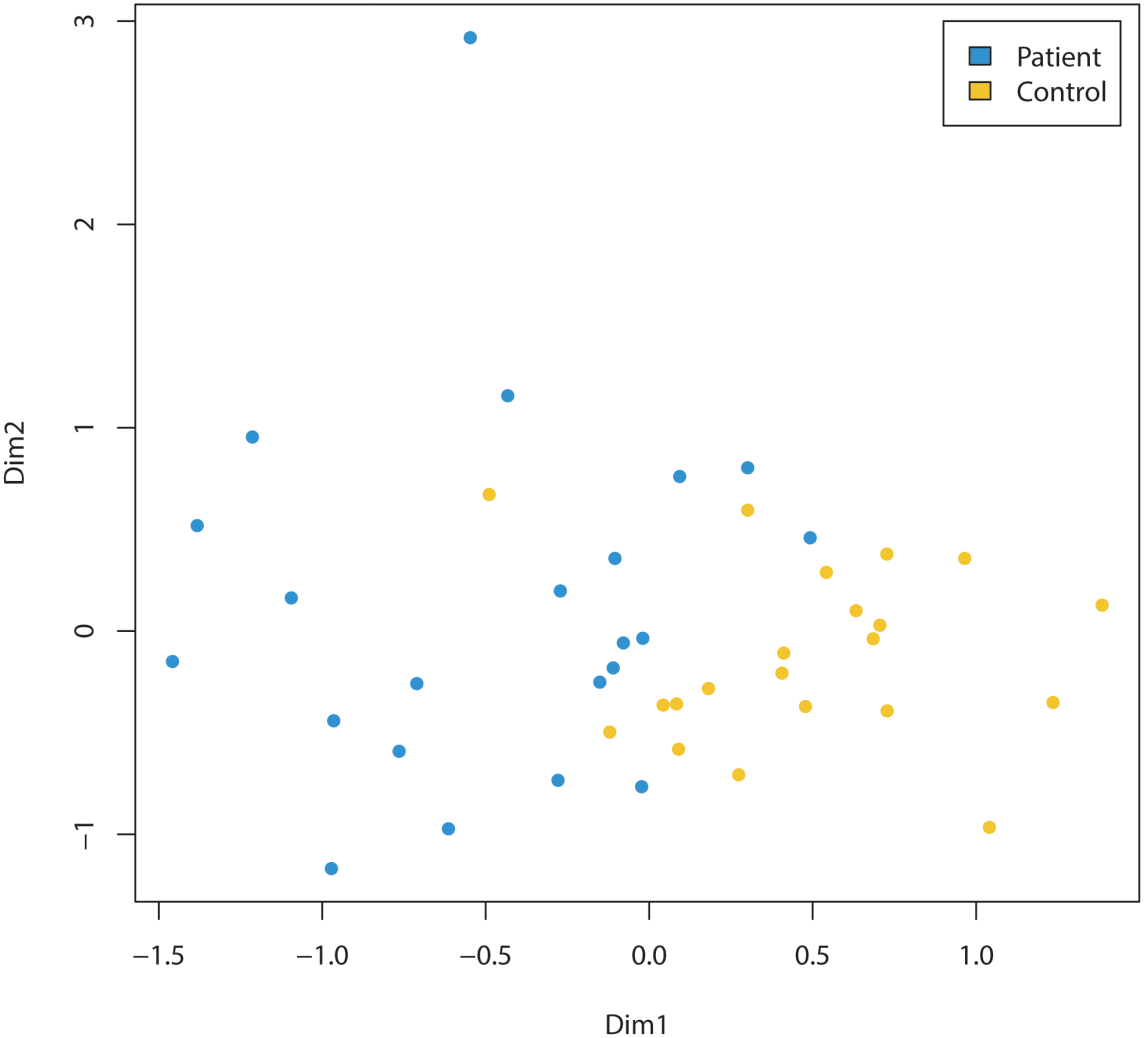
Comparison of the microbiomes of children (three years of age and older) diagnosed with cancer (Patient) and healthy (Control) children (three years of age and older). Non-metric multidimensional scaling of amplicon sequence variants (ASVs) relative abundance data, based on Bray-Curtis between-sample distances in children diagnosed with cancer (=patients, indicated by blue dots) compared to control cohort of children sampled from the kindergartens (=controls, indicated by yellow dots). The microbiota composition was significantly different (R^2 = ^0.09, p<0.001, PERMANOVA) between the two groups.

Children diagnosed with cancer had a significantly different microbiota composition (R^2 ^= 0.09, p<0.001, PERMANOVA) compared to the microbiotas of the cohort of children sampled from the kindergartens (**Figure 1**). This result was confirmed by a machine learning approach using a random forest classification model. This resulted in a classification accuracy of 100.0%, with no misclassified samples out of 43 (p<0.001, permutation test). It should be noted that inclusion of children two years of age in both the patient and the healthy cohort did not alter the discrimination of the microbiotas between the groups (R^2 ^= 0.09, p<0.001, PERMANOVA).

We observed a marginally higher Shannon entropy in the patient group relative to controls (p<0.09, Wilcoxon rank sum test, **Figure S3B**), while ASV richness did not differ significantly between the groups (**Figure S3A**). At the phylum level, we observed an increased mean relative abundance of Actinobacteria in the patient group (p=0.042, Wilcoxon rank sum test) (**Figure S4**), but no other significant differences.

**Figure 2:**
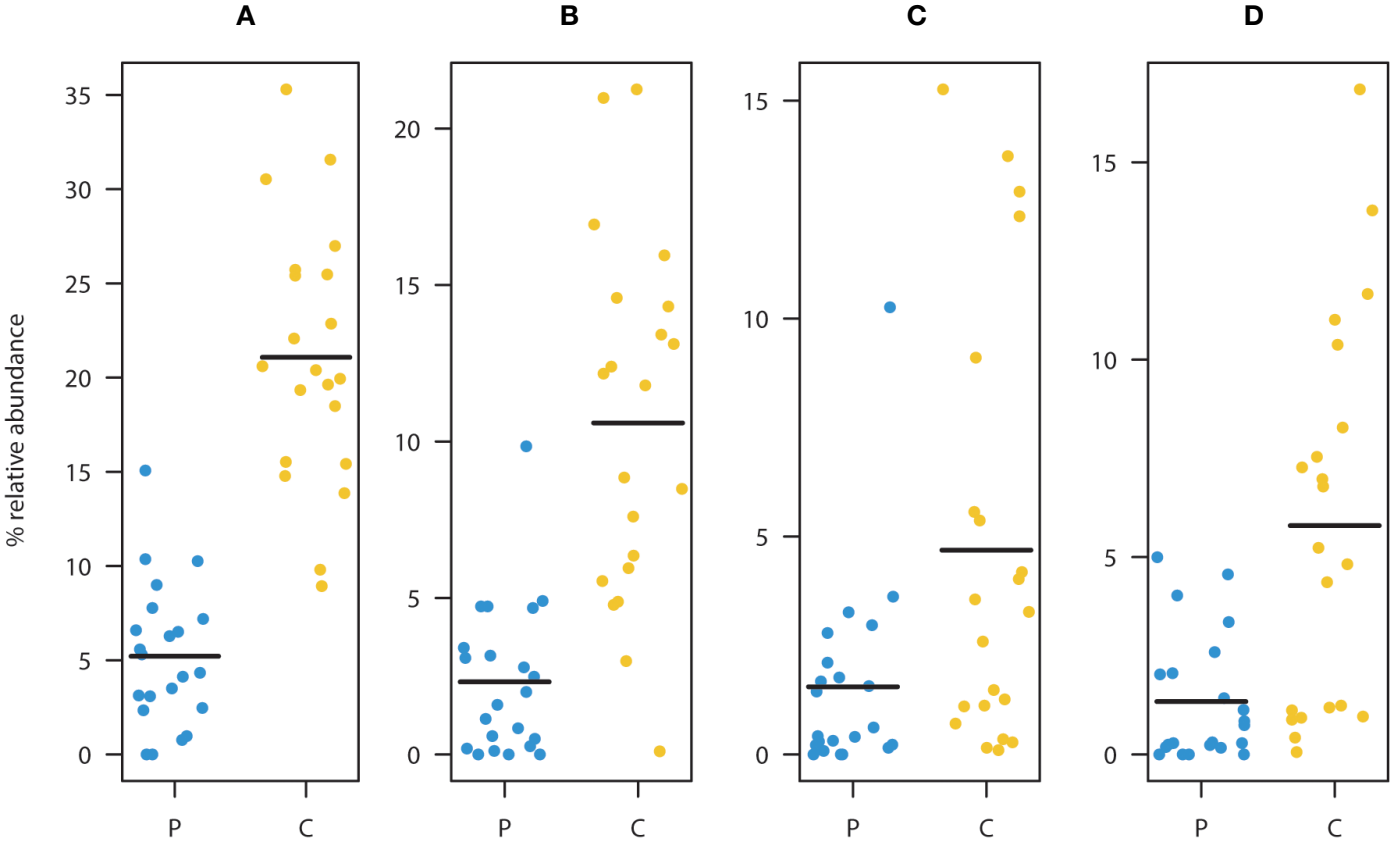
Comparison between patients (P) and controls (C) of the relative abundance of ASV’s assigned to as *Faecalibacterium prausnitzii* **(A)**. Summed relative abundances of ASV2, ASV3, and ASV6 (main taxa assigned to *F. prausnitzii*). Relative abundances of ASV2 **(B)**, ASV3 **(C)**, and ASV6 **(D)**. These ASVs were classified as *F. prausnitzii* and were found to be significantly depleted in the patient group relative to the healthy controls. (Black lines represent means, yellow dots represent the individual relative abundances of the controls three years of age and older, and blue dots represent the individual relative abundances in the children three years of age and older in the diagnosed group).

ASV level analysis using DESeq2 identified 13 ASVs with significantly (Benjamini-Hochberg corrected p<0.05) differential occurrence between the patient and the control group (**Table 2**). six of these ASVs were depleted in children diagnosed with cancer. Strikingly, three of these eight were classified as *Faecalibacterium prausnitzii* (ASV2, ASV3, and ASV6). The combined relative abundance of these three ASVs were significantly higher in the control cohort (p<<0.001) (**Figure 2A**). All three groups were highly prevalent in the data (**Figures 2B–D**) and additional testing confirmed the DESeq2 results (Wilcoxon rank sum test ASV2=p<0.0001, ASV3 p<0.012, and ASV6 p<0.001). In order to investigate the robustness of our results if the young children were included, we checked that no significant relationship was observed between the relative abundance of these three ASVs and the age parameter in the patient group (**Figures S5A–C**). Furthermore, all three ASVs were found in both the patients and the controls. Since systematic bias could be introduced by differences in sample collection, we compared the combined relative abundances of ASVs assigned to *F. prausnitzii* in fecal samples that were frozen (7 samples, 3 individuals) with paired samples collected in ethanol (7 samples, 3 individuals). No statistically significant difference was observed (Wilcoxen rank sum test p=0.32). The other three ASVs depleted in patients were *Haemophilus parainfluenzae*, *Sutterella wadsworthiensis*, as well as *Ruminococcus*. Among the ASVs that were significantly overrepresented in the patient group, were *Prevotella copri*, *Steptococcuss*, and *Dorea longicatena*.

**Table 2:** Amplicon sequence variants (ASVs) level analysis using DESeq2 identified 11 ASVs with significant differential occurrence between the patient and the control group (Benjamini-Hochberger corrected p<0.05).

Taxonomic assignment	Mean count	logFoldChange	P-value
Faecalibacterium prausnitzii (ASV2)	3852.29	-2.94	0.000001
Haemophilus parainfluenzae (ASV64)	107.29	-3.06	0.000018
Faecalibacterium prausnitzii (ASV6)	1973.35	-2.88	0.000026
Faecalibacterium prausnitzii (ASV3)	2765.53	-2.74	0.000583
Prevotella copri (ASV20)	52.45	3.75	0.005837
Alistipes finegoldii (ASV17)	780.24	-2.60	0.008112
Clostridium_IV (ASV30)	503.94	-3.96	0.018791
Clostridium_XlVa (ASV46)	82.17	3.24	0.029148
Sutterella wadsworthensis (ASV38)	424.90	-3.17	0.048519
Dorea longicatena (ASV88)	103.83	1.62	0.048519
Clostridium_XlVa (ASV145)	56.50	-1.97	0.048519

ASV2, ASV3, and ASV6 were also variables of high importance for successful discrimination between the patients and the controls in the random forest model (**Figures S6A, B**). The random forest model also identified the following short chain fatty acid producing taxa as being among the top 30 variables of highest importance for successful classification: *Roseburia* spp., *Ruminococcus* spp., *Anaerostipes hadrus*, *Coprococcus comes, Intestinimonas*, *Lachnospiracea*, *Roseburia inulinivorans*, and *Blautia faeces*.

## Discussion

### Outline of the study

We characterized the fecal microbiota in a cohort of children diagnosed with cancer prior to the beginning of treatment in relation to 25 healthy children attending kindergarten. The cohort of patients included children ranging from newborn to 17 years of age. While the overall age effect within that cohort was large, excluding children younger than three years removed this effect. Previous work has shown that the gut microbiota tends to stabilize at an adult-like stage by three years of age (Yatsunenko et al., 2012; Stewart et al., 2018). Thus, while not optimal, removing the youngest children from the patient cohort should largely account for age differences relative to controls. We would like to point out that the present study is a small pilot, with imperfect age matching between patients and controls, and the results presented here should be substantiated in larger scale studies.

### Differences in the sampling procedure

Sampling procedures were different in the patient and the control group. Specifically, the samples from the kindergarten cohorts were collected at home by the parents or at kindergarten by the teachers into tubes containing 95% ethanol. Samples from the cohort diagnosed with cancer were collected at the hospital and frozen directly upon sampling, without any kind of preservation medium. In a study analyzing 1200 samples, the authors found that these preservation methods did not significantly interfere with the biological signature of the samples (Song et al., 2016). A second study by Moossavi et al. (2019), found consistent microbiota profiles in samples preserved in ethanol and by freezing. This study also did not observe discernible differences in *Faecalibacterium* spp. abundances. Similar conclusions have been drawn in other studies (Blekhman et al., 2016; Byrd et al., 2019; Marotz et al., 2021).

### Decreased relative abundance of *Faecalibacterium prausnitzii* in the diagnosed cohort

The most striking result was the depletion of three abundant ASVs classified as *F. prausnitzii* in children diagnosed with cancer (**Figure 2**). The depletion was highly significant for all three strains and overall *F. prausnitzii* was decreased by a factor of four in patients relative to the controls. *F. prausnitzii* is a dominant member of the human microbiota and a lowered relative abundance can correlate with negative health outcomes, like inflammatory bowel disease and colorectal cancer (Lopez-Siles et al., 2016; Lopez-Siles et al., 2017). Due to its high butyrate production and anti-inflammatory properties, it is considered to present a promising candidate for probiotic formulations (Ferreira-Halder et al., 2017). Short chain fatty acids (acetate, propionate and butyrate) (*Reviewed* in Blaak et al., 2020) are produced by bacteria in the colon and are important in vital gut processes, like gastrointestinal motility and energy utilization. For example, butyrate is the main nutrient source for colonocytes and it has been shown to have protective properties against colorectal cancer (Archer et al., 1998). Furthermore, butyrate can reduce inflammation of the intestinal mucosa through regulation of immune signaling (*Reviewed* in Deleu et al., 2021). *F. prausnitzii* is one of the most important butyrate producers in the gastrointestinal tract and thus of high interest in biomedical research. Modulation of this species, as well as other short chain fatty acid (SCFA) producers, may have consequences for treatment efficacy in cancer. Here in this study, several other SCFA producing taxa were identified as important for classification of microbiota profiles into the patient and the control group.

Our results indicate that changes in the microbial community involved in SCFA production in the gut may function as an early indicator of the initiation or presence of cancer. In particular, the butyrate producing *F. prausnitzii* was depleted by a factor of four in the patient group. *F. prausnitzii* reduction has also been reported in gastrointestinal conditions, like inflammatory bowel disease (Zhang et al., 2016), indicating that this species could be a more general indicator of disease-associated dysbiosis. From the data presented here, it is unclear whether *F. prausnitzii* depletion results from cancer, or if a dysbiotic microbiome can actually play a role in the development of disease. While the main results from this pilot study seem quite robust, it should be noted that there are clear limitations to this study that may affect the generalizability of the conclusions. Childhood cancer is relatively rare and study recruitment is challenging. This has affected the scale of this study and has implications for the statistical power. Furthermore, samples were collected from patients at a single pediatric ward which could also introduce bias due to a shared sampling environment. Lastly, although we were unable to detect differences in *F. prausnitzii* relative abundances due to sample storage methods, this can a source of bias and future studies should strive to have a more consistent sampling regime. Other factors are known to influence the composition of the gut microbiome and should be addressed in the design of future, larger studies, including questionnaires about cancer symptoms at diagnosis, body mass index and diet.

## Data availability statement

The datasets presented in this study can be found in online repositories. The names of the repository/repositories and accession number(s) can be found below: https://www.ncbi.nlm.nih.gov/genbank/, PRJNA814879.

## Ethics statement

The studies involving humans were approved by Regional Committee for Medical and Health Research Ethics (REK nr: 2018/2491 and 2019/66945). The studies were conducted in accordance with the local legislation and institutional requirements. Written informed consent for participation in this study was provided by the participants’ legal guardians/next of kin.

## Author contributions

LB and MM-K initiated the overall study. LB and MM-K conceived and designed the childhood cancer, EM, PT, NN, and PF the control study. EM, PT, NN, PF, VS, and JH tested and established various methods for the study. NR and JH collected, prepared and managed the samples from children diagnosed with cancer, EM, NN, and PF from children of the control study from Norwegian kindergartens. EM, PT, and VS performed DNA extraction and sequencing. EM and PT established the analysis pipeline and carried out the data analysis. EM, PT, MM-K, and LB interpreted the data. EM and PT wrote the draft of the manuscript, JH, MM-K, NN, and PJ critical reviewed the manuscript, and EM and LB finalized it. All authors read and approved the final manuscript. All authors contributed to the article.
