# Supplementary material for: Reduced abundance of Faecalibacterium prausnitzii in the gut microbiota of children diagnosed with cancer, a pilot study
Source: Front Microbiomes. 2023 Nov 7;2:1151889. doi: 10.3389/frmbi.2023.1151889 (PMC12993630; doi:10.3389/frmbi.2023.1151889)
Supplement: Supplementary file 1 [file DataSheet_1.docx]

**Supplementary material for “Reduced abundance of *Faecalibacterium prausnitzii***

**in the gut microbiota of children diagnosed with cancer, a pilot study”**

**Supplementary figures S1-S6**

**Abbreviation**: ASV, amplicon sequence variant and NMMS, non-metric multidimensional scaling


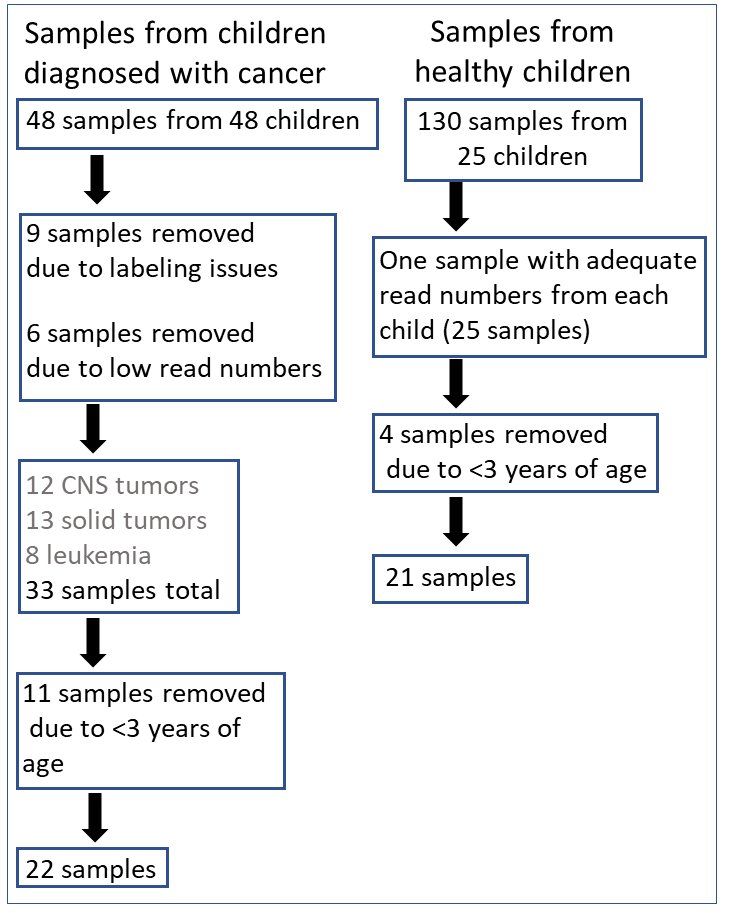


**Supplementary figure 1.** **Outline of samples in the study.** Fecal samples from children diagnosed with cancer were collected prior to treatment at the hospital. Samples were then withdrawn from the Norwegian Childhood Cancer Biobank (NCCB), where biological samples are stored according to standard protocols (Hermansen et al., 2021). After removal of samples due to labeling issues and low read counts, 33 patients remained. Of these, 12 had been diagnosed with tumors of the central nervous system (CNS), 13 with solid tumors (ST), and eight with leukemia (**Table 1**). 11 samples were removed from comparisons due to children being <3 years of age. Fecal samples from healthy children were collected at home by the parents or at kindergarten by the teachers.

**
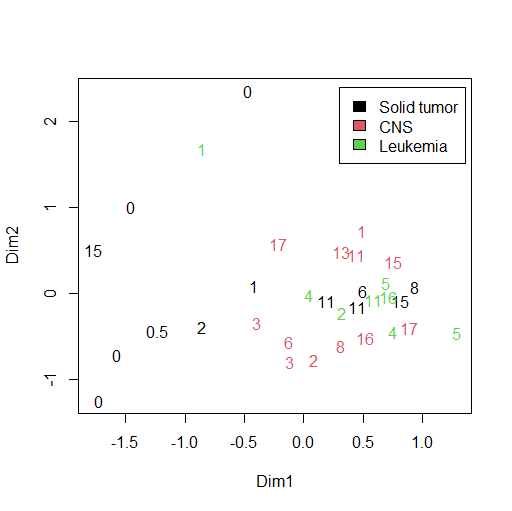
**

**Supplementary figure 2.** Non-metric multidimensional scaling (NMMS) of amplicon sequence variants (ASVs) relative abundance data, based on Bray-Curtis between-sample distances, for the entire patient group. In the plot, each sample is represented by a number indicating the age (in years) of each patient at the time of sampling. The specific diagnosis is indicated by color.

**|
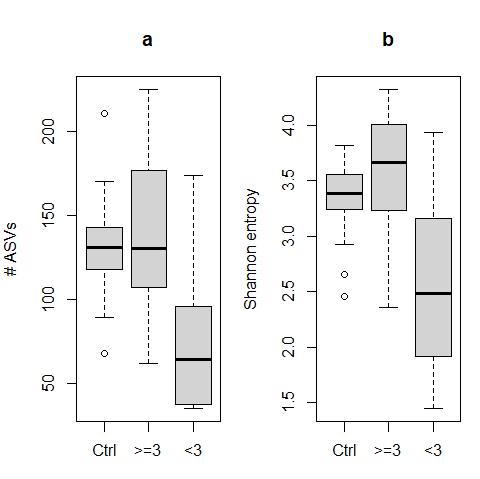
**

**Supplementary figure 3**. ASV richness (a) and Shannon entropy (b) in the healthy control group (Ctrl), patients three years or older (>=3), and patients younger than three years (<3). (Black lines inside boxes are medians, boxes represent the interquartile range, and the whiskers represent 1.5 times the interquartile range).

**
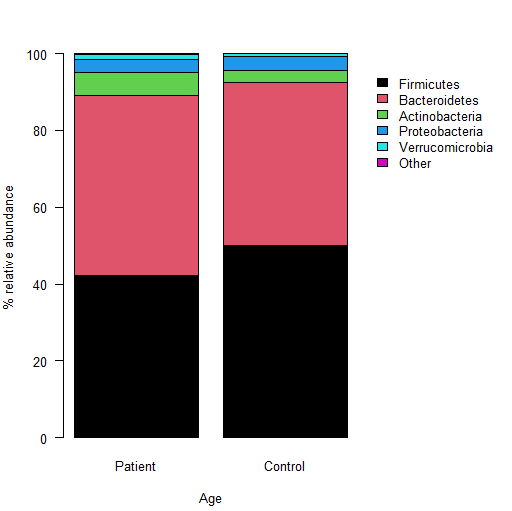
**

**Supplementary figure 4.** Relative abundance of the main bacterial phyla in the patient (three years or older) and the control group.


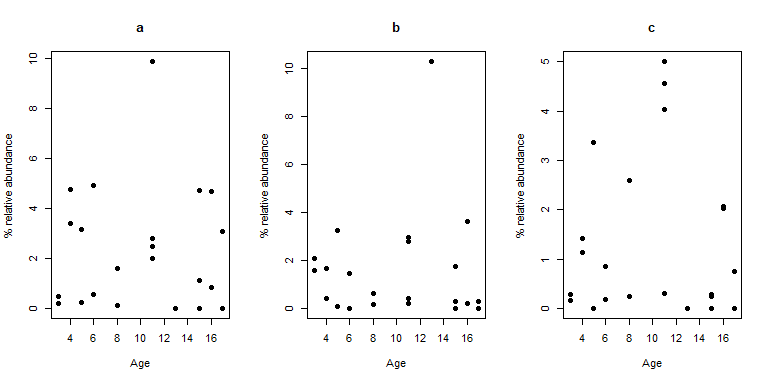


**Supplementary figure 5**. Relative abundances of ASV2 (a), ASV3 (b), and ASV6 (c) as a function of age in the patient group. These ASVs were classified as *F. prausnitzii* and were found being significantly depleted in the patient group relative to the healthy controls. There was no significant relationship between abundance and age for any of these ASVs (linear models).


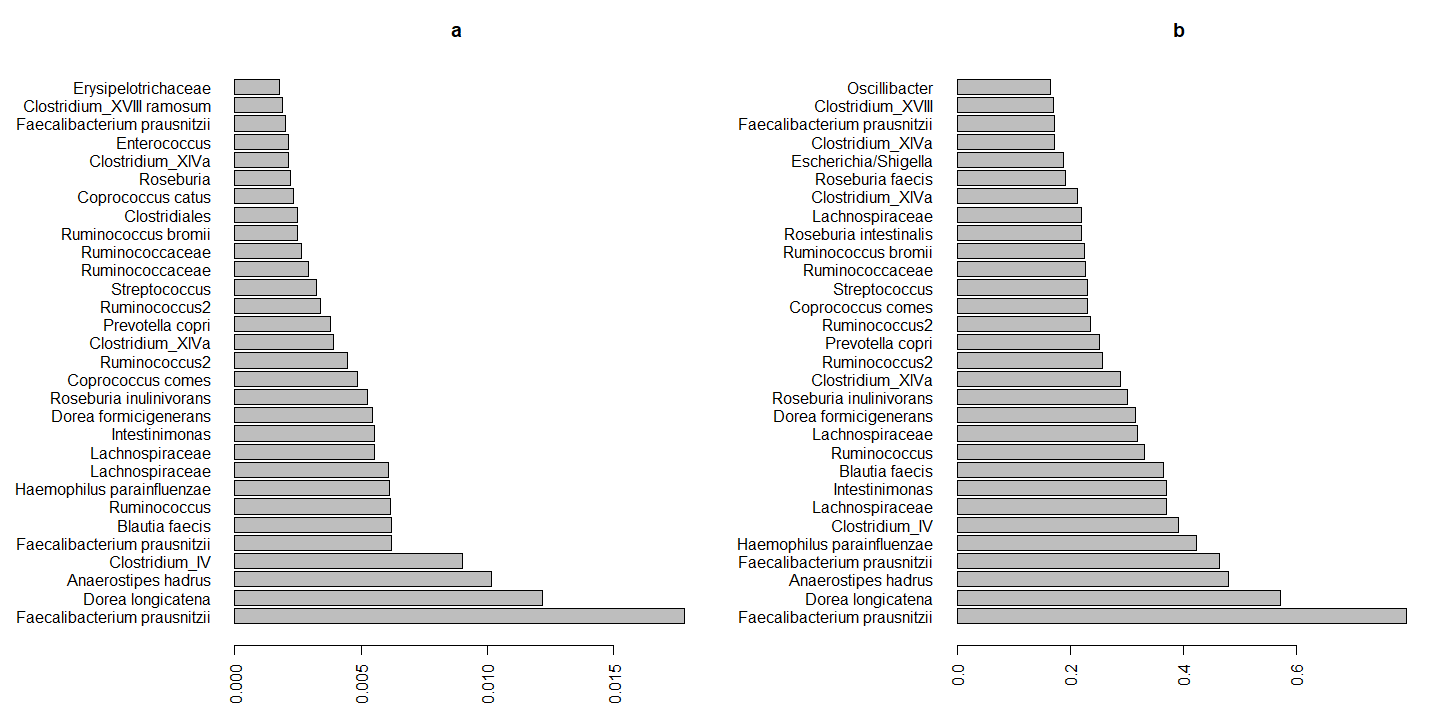


**Supplementary figure 6**. Top thirty most important variables (ASVs) for discriminating between patients and controls in the Random forest classification model, as measured by (a) mean decrease in accuracy and (b) mean decrease in Gini coefficient. In (a) the ASVs classified as *F. prausnitzii* are ASV2 and 6, respectively (from most to less important). In (b) the ASVs classified as *F. prausnitzii* are ASV2 6 and 3, respectively.
